# A new assessment model for tumor heterogeneity analysis with [18]F-FDG PET images

**DOI:** 10.17179/excli2015-723

**Published:** 2016-01-28

**Authors:** Ping Wang, Wengui Xu, Jian Sun, Chengwen Yang, Gang Wang, Yu Sa, Xin-Hua Hu, Yuanming Feng

**Affiliations:** 1 Department of Radiation Oncology, Tianjin Medical University Cancer Institute and Hospital, Tianjin, China; 2 Department of Molecular Imaging and Nuclear Medicine, Tianjin Medical University Cancer Institute and Hospital, Tianjin, China; 3 Department of Biomedical Engineering, Tianjin University, Tianjin, China; 4 Department of Physics, East Carolina University, Greenville, NC

**Keywords:** [18]F-FDG PET image, standard uptake value, intratumor heterogeneity, gray level co-occurrence matrix

## Abstract

It has been shown that the intratumor heterogeneity can be characterized with quantitative analysis of the [18]F-FDG PET image data. The existing models employ multiple parameters for feature extraction which makes it difficult to implement in clinical settings for the quantitative characterization. This article reports an easy-to-use and differential SUV based model for quantitative assessment of the intratumor heterogeneity from 3D [18]F-FDG PET image data. An H index is defined to assess tumor heterogeneity by summing voxel-wise distribution of differential SUV from the [18]F-FDG PET image data. The summation is weighted by the distance of SUV difference among neighboring voxels from the center of the tumor and can thus yield increased values for tumors with peripheral sub-regions of high SUV that often serves as an indicator of augmented malignancy. Furthermore, the sign of H index is used to differentiate the rate of change for volume averaged SUV from its center to periphery. The new model with the H index has been compared with a widely-used model of gray level co-occurrence matrix (GLCM) for image texture characterization with phantoms of different configurations and the [18]F-FDG PET image data of 6 lung cancer patients to evaluate its effectiveness and feasibility for clinical uses. The comparison of the H index and GLCM parameters with the phantoms demonstrate that the H index can characterize the SUV heterogeneity in all of 6 2D phantoms while only 1 GLCM parameter can do for 1 and fail to differentiate for other 2D phantoms. For the 8 3D phantoms, the H index can clearly differentiate all of them while the 4 GLCM parameters provide complicated patterns in the characterization. Feasibility study with the PET image data from 6 lung cancer patients show that the H index provides an effective single-parameter metric to characterize tumor heterogeneity in terms of the local SUV variation, and it has higher correlation with tumor volume change after radiotherapy (R^2^ = 0.83) than the 4 GLCM parameters (R^2^ = 0.63, 0.73, 0.59 and 0.75 for Energy, Contrast, Local Homogeneity and Entropy respectively). The new model of the H index has the capacity to characterize the intratumor heterogeneity feature from 3D [18]F-FDG PET image data. As a single parameter with an intuitive definition, the H index offers potential for clinical applications.

## Introduction

Tumor microenvironment has demonstrated heterogeneities which include variations which include variation in degree of vascularity, hypoxia, proliferation rates, metabolic rates, and gene expression (Eary et al., 2008[5]; Kidd and Grigsby, 2008[10]). It has been shown that the heterogeneity in intratumor metabolism correlates strongly with tumor lymph node metastasis, radiation sensitivity, local recurrence and survival rates. The heterogeneity affects also, to a certain degree, the patient's prognosis and response to treatment (Kidd and Grigsby, 2008[10]; Tixier et al., 2011[16]). Therefore, quantitative study of intratumor heterogeneity can yield critical insights on cancer diagnosis and treatment planning. This leads to increased research interests on development of new modeling tools for quantification of the heterogeneity within the tumor tissues (Tixier et al., 2011[16]).

As a functional imaging method, positron emission tomography (PET) provides physiological information of tumors (Schiepers and Dahlbom, 2011[14]; Czernin et al., 2007[3]; Belhassen and Zaidi, 2010[1]). The [18]F fluorodeoxyglucose PET ([18]F-FDG PET) is useful to gauge the metabolic activities of tumors because most malignant tumor cells have high glucose metabolic rates (Schiepers and Dahlbom, 2011[14]). An [18]F-FDG PET image can be utilized to obtain the standard uptake value (SUV) of the radiotracer either voxel-wise or over a region of interest (ROI) which serves as a metric of glucose metabolism within the tumor (Tixier et al., 2011[16]). For example, it has been reported that intratumor heterogeneity can be characterized with quantitative analysis of the [18]F-FDG PET image data (Eary et al., 2008[5]; Tixier et al., 2011[16]). Most of the published models are for the assessment of intratumor heterogeneity from the molecular biology point of view, which describes the tumor growth or metabolism dynamics (Eary et al., 2008[5]; O'Sullivan et al., 2005[12]; Wu et al., 1995[17]; Li et al., 2010[11]; Gonzalez-Garcia et al., 2002[8]; Geisler et al., 2002[7]). Among these works some studies adopted an image texture based approach in extracting intratumor heterogeneity information from the pre-therapy PET images of patients (Tixier et al., 2011[16]; El Naqa et al., 2009[6]). El Naqa and colleagues used the textural features to predict treatment outcomes from baseline [18]F-FDG PET images of patients with cervical and head-and-neck cancers. In their study, the methods of intensity-volume histogram, geometrical shape features and gray level co-occurrence-matrix (GLCM) parameters of energy, contrast, local homogeneity and entropy were used for the characterization of [18]F-FDG uptake heterogeneity in tumor or ROI (El Naqa et al., 2009[6]). Tixier and colleagues verified the effectiveness of the GLCM parameters in assessing intratumor heterogeneity and studied their predictive value for the response of esophageal cancer patients to radiochemotherapy (Tixier et al., 2011[16]). Tan and colleagues used 19 histogram distances to quantitatively analyze longitudinal patterns of [18]F-FDG uptake in tumor and concluded that the patterns characterized using 14 histogram distances provide useful information for predicting the pathologic response of esophageal cancer to neoadjuvant chemoradiotherapy (Tan et al., 2013[15]).

The existing models employ multiple parameters for feature extraction which makes it difficult to implement in clinical settings for quantitative characterization of tumor cell response to radiotherapy. A simple, self-sufficient and easy-to-use mathematical model can be very helpful to utilize intratumor heterogeneity information extracted from [18]F-FDG PET image data for cancer diagnosis, staging, treatment planning, prognosis and response assessment in routine cancer management. For this purpose we have developed a new model for intratumor heterogeneity assessment with a single-parameter index as a quantitative tool for future clinical studies. The model has been tested and validated with analytical spherical phantoms and patients' [18]F-FDG PET image data to evaluate its effectiveness. Our results demonstrated that the new model can serve as an objective descriptor of intratumor heterogeneity. We present the model in the next section followed with results and discussion.

## Materials and Methods

SUV is defined as a measurement of activity per unit volume of tissue normalized by the administered radiotracer activity per unit of body mass at the time of image acquisition. Hence SUV depends on the initial FDG uptake kinetics and radiotracer distribution which in turn are functions of the initial dose and elapsed time between injection and image acquisition. Based on these considerations we have developed the following guidelines for development of the new model. First, the single-parameter index should be related to the differential distribution of the voxel-wise SUV in the tumor for quantification of heterogeneity. Secondly, simple and easy-to-understand definition is preferred since it is intended for clinical implementations. Last the model should reflect clinical evidence that peripheral heterogeneity often indicates diffusing tumors of poor prognosis (Cao et al., 2009[2]; Owonikoko et al., 2002[13]).

With these guidelines a dimensionless parameter of H index is defined to characterize the heterogeneity and direction of SUV variation within the tumor. By summing the local difference of SUV among the voxels within the tumor or ROI, and size-weighted index of heterogeneity is defined as


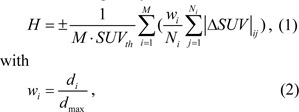


where *M is th*e number of voxels within the segmented tumor, *N_i_ is the number of voxels within the tumor adjacent to the ith voxel which is 26 or less, |ΔSUV|_ij_ is *the absolute value of SUV difference between the ith and its jth adjacent voxel, *SUV_th _is a threshold value of SUV for segmenting the tumor*,* d_i_ is t*he distance of ith voxel from SUV-based tumor center at **r**_c_ as discussed below and *d_max_* is the maximum value of *d_i_ for I = 1, 2, …, M*. The SUV-based tumor center or simply tumor center is defined by **r**_c_ = (x_1c_, x_2c_, x_3c_) in *the following with *SUV_i_ and x_ni_ as the SUV and nth coordinate of the ith voxel, respectively


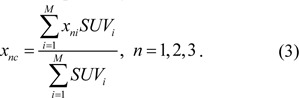


In the definition of H given by Eq. (1) the weight factor w_i_ is employed to increase the contribution of the peripheral heterogeneity associated with the voxel i and the sign of ± is used to indicate a descending (+) or ascending (-) variation of averaged SUV from the tumor center to the periphery which has certain clinical implications (Cao et al., 2009[2]; Owonikoko et al., 2002[13]). To determine the sign we calculate two times for the tumor, the first calculation uses the whole tumor volume, the second calculation uses the volume shrunk from the periphery to the center by 1/2 to get averaged SUV for the inner part of the tumor. Then the sign of H index is determined by comparing the two averaged SUV values, if the first value is less than or equal to the second one, a (+) sign is given to the H index to indicate the descending change from the center to the periphery, otherwise a (-) sign will be given to the H index. SUV_th_ can be set by users and was assumed to be 2.5 (g/ml) for our study. With the above procedure the H index is a dimensionless parameter and its magnitude correlates positively with the extent of heterogeneity in the spatial distribution of SUV or [18]F-FDG PET image data. 

To investigate the relation of the H index with the image textures, we selected the GLCM algorithm to quantify the textures. GLCM has been used widely as a powerful tool for analysis of image textures for its capacity to identify second-order spatial relationships between pixels or voxels of the input image data by constructing matrix P (Haralick, 1979[9]; Dong et al., 2011[4]). The matrix elements P (g, h; d, a) are determined by the co-occurrence probability of two gray levels or intensities of g and h at two neighboring voxels separated by a pre-determined vector of distance d and direction a. Thus the row and column positions of an element are given by the selected gray levels and, therefore, GLCM can be presented as a square image with a size equal to the number of gray levels G. Once the GLCM image is obtained, multiple statistical parameters can be derived for quantification of the input image textures. In our study the distance vectors of (d, a) were set to cover all of the nearest neighbors for each selected voxel in three-dimensional (3D) or 2D space whose maximum values is 26 or 8 respectively with d=1. After the calculation of P for an input [18]F-FDG PET image, 4 parameters of Energy, Contrast, Local Homogeneity and Entropy were obtained to quantify the image textures. The definitions of these GLCM parameters are given below (El Naqa et al., 2009[6]; Haralick, 1979[9]; Dong et al., 2011[4]).


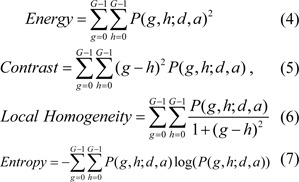


Among the above parameters, Energy is a measure of image uniformity whose larger value indicates a more uniform and regular changing texture pattern. Contrast is proportional to the variations of gray levels present in the input image and favors contributions from the elements of P matrix away from the diagonal or large variations in gray levels while Local Homogeneity provides a similar measure but favoring those elements close to the diagonal. Finally Entropy measures the randomness of intensity distribution and reaches its maximum value if all P elements are of random values. It should be noted that these parameters are independent of the position, orientation, size, and gray levels of the tumor in the input image and take into account only the local spatial distribution of gray levels.

**Figure 1:**
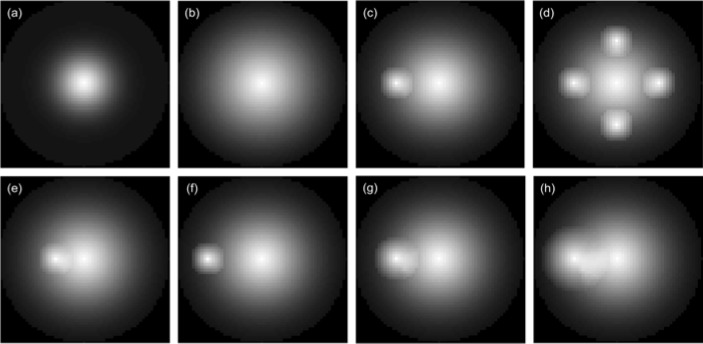
Cross-sectional views of selected 3D spherical phantoms, (a) and (b): single modified Gaussian spheres of size parameter R=4 and 12; (c) and (d): multi-sphere assemblies with variable number of smaller spheres N=1 and 4, the radius of the smaller sphere r_s_=6 and the distance between the centers of large and smaller spheres D=15; (e) and (f): two-sphere assemblies with variable center distance D=10 and 19 and r_s_=6; (g) and (h): two-sphere assemblies with variable radius r_s_=8 and 12 and D=15. For all images SUV_max_ is set to 40 for all cases and for images (c) to (h) the size parameter R of the large sphere is set to 30 in the unit of voxels.

**Figure 2:**
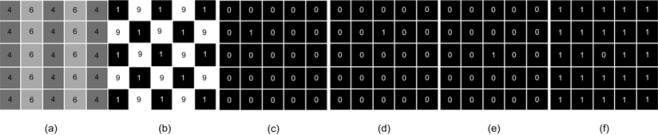
2D phantoms with different SUV distributions

To validate the new heterogeneity assessment model, two types of phantoms with different distributions of gray levels assumed as SUV were created and analyzed using the Matlab software (MathWorks, Inc., Natick) for calculations of the GLCM parameters and H index. The first type of phantoms consists of either single spheres or multi-sphere assemblies representing 3D tumors without or with heterogeneous sub-regions of high SUV inside the tumors to mimic the clinical situations. The large sphere has a radius of r_max_=30 voxels and the variation of SUV or the voxel intensity is of a modified Gaussian profile with the center peak value fixed at 40 (=SUV_max_) and 2.0 (g/ml) (=SUV_th_) at the surface. The following equation defines SUV at **r**


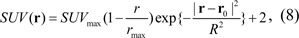


where **r**_0_ is the sphere center and R is an adjustable size parameter for the Gaussian function. The SUV distributions of small spheres also follow similar modified Gaussian profiles with its center peak values fixed at 40 and reduced along their radii to values at the surfaces which are the same as the SUV value of their surrounding voxels. Different cases were considered which include single spheres of various R values, multi-sphere assemblies with different number of small spheres representing sub-regions of high SUV, and two-sphere assemblies with a small sphere of different location (D) and radius (r_s_) inside the large sphere phantom. Figure 1(Fig. 1) presents the cross-section views of these phantoms. A homogeneous image (R=0) with all voxels having the same SUV of 2.0 (g/ml) was used for baseline comparison. The H index and GLCM feature parameters were calculated and compared for all phantoms to evaluate the new model. In addition to the 3D spherical phantoms, we also employed second type of 2D phantoms of six SUV distributions to demonstrate the difference between the H index and GLCM parameters on characterization of SUV distributions. The 2D phantoms are displayed in Figure 2(Fig. 2).

**Figure 3:**
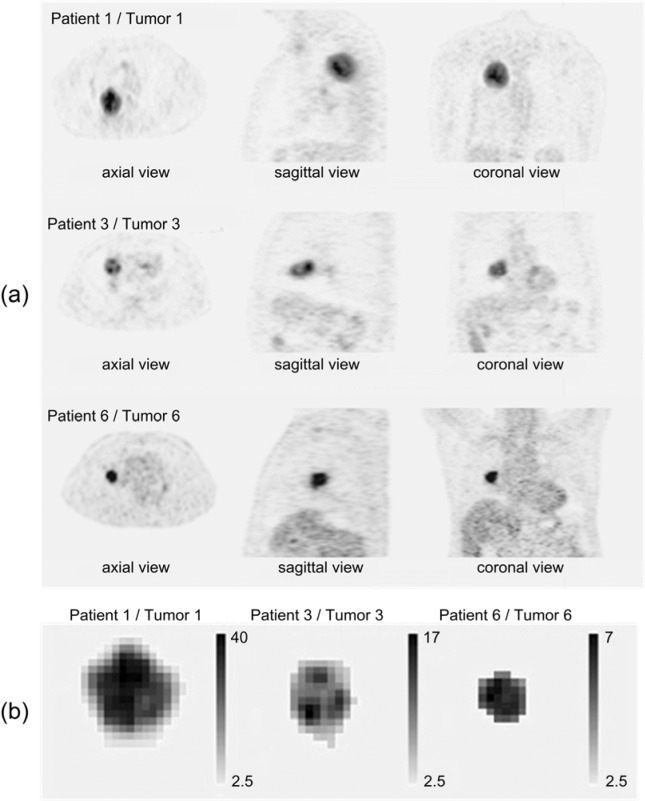
The [18]F-FDG PET images of three lung cancer patients with one tumor per patient, (a) different cross-sectional views; (b) the tumors segmented from the images in (a).

We also applied the H index on [18]F-FDG PET image data from 6 non-small cell lung cancer (NSCLC) patients to test the feasibility of the new model for future research and application in clinics. The images were acquired from the patients before their radiotherapy or radiochemotherapy with a PET scanner (Discovery ST, General Electric, Inc., New York). The PET image processing software MIM 5.2 (MIM Software, Inc., Cleveland) was used to determine the voxel-wise distribution of SUV in the PET images and the tumor regions were segmented with the definition of SUV_th_ = 2.5 (g/ml). Subsequently the H value and GLCM texture parameters were calculated within the tumors. Three patients' PET images and the segmented tumor regions are shown in Figure 3 (a) and (b)(Fig. 3) as examples. To investigate correlation of the H index and GLCM parameters with tumor response to radiation treatment, post-treatment change of tumor volume, which is the important indicator of the response, was measured with the planning CT image and follow-up CT image at the time of 4 months after treatment for each patient. The percentage differences between post and pre-treatment volumes defined as the ratio of volume change to pre-treatment volume were recorded. Correlation determination R^2^ was used in the analysis.

## Results and Discussion

**Figure 4:**
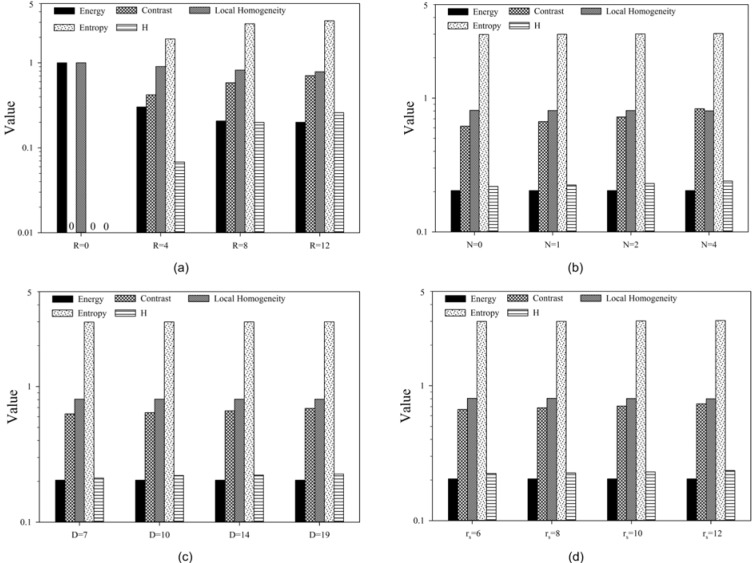
GLCM parameters and H index obtained from the 3D spherical phantoms. (a): single spheres of different size parameter R, zero represents an invisible bar; (b): multi-sphere assembly with small spheres of variable number N; (c): two-sphere assembly with small sphere of variable distance D; (d): two-sphere assembly with small sphere of variable radius r_s_

**Figure 5:**
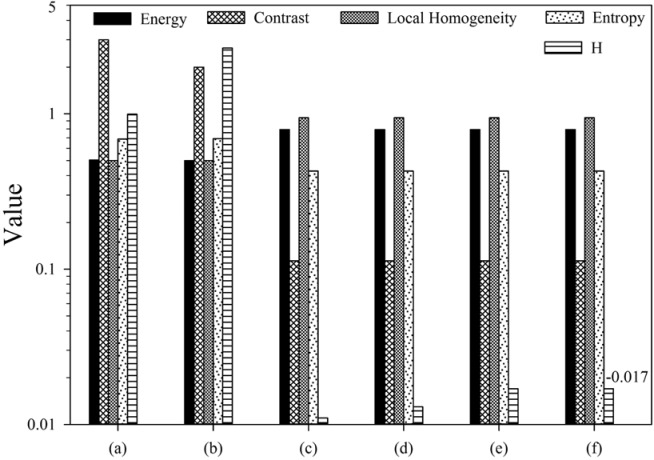
The GLCM parameters and H index obtained from the 2D phantoms shown in Figure 2, the H bar in (f) is marked with its negative value of -0.017

Model validation was first performed with the 3D phantoms and 2D phantoms followed by a feasibility study on the patients' image data. The H index and GLCM parameters were obtained from spherical phantoms of different degrees of heterogeneity to investigate the relation between the H index and the textures of the 3D images. The GLCM parameters were calculated and compared to the H index for each phantom shown in Figure 1(Fig. 1) with results presented in Figure 4(Fig. 4). The results of parameter comparison using the six 2D graphics are displayed in Figure 5(Fig. 5). The data clearly show that for these 2D cases the H index can provide a measure of the heterogeneity within the phantoms than the selected GLCM parameters. In Figure 2(Fig. 2), it can be seen that image (a) and (b) have the same averaged SUV value of 5 but obviously very different distributions with image (b) more heterogeneous than (a). Figure 5(Fig. 5) demonstrates that the H index for (b) is about 2.7 times higher than that for (a) while only the Contrast among the 4 GLCM parameters exhibits variation with the value for (a) 1.5 times higher than the value for (b). For image (c) through (f), one can find they are of different SUV distributions and the H index can clearly differentiate these images but the 4 GLCM parameters remain the same for them respectively and fail to differentiate them.

The availability of an effective single-parameter index to characterize tumor heterogeneity can benefit substantially the clinical treatment and management of cancer patients. To achieve this goal we have selected image data of [18]F-FDG PET as the primary source of information for modeling of the heterogeneous metabolic activities within tumors. A single dimensionless parameter of H index has been defined and obtained from the 2D and 3D distribution of SUV as a parameter for characterization of tumor heterogeneity. Through the validation study with the 3D spherical phantoms we found that the H index increases in each case of 4 single-sphere phantoms with increasing R (see Figure 4(a)(Fig. 4)) as expected, except the baseline case of R= 0 of homogeneous SUV distribution. However, the rate of the H index increase becomes much less in the other 3 types of spherical phantoms as shown from Figure 1(c) to 1(h)(Fig. 1) with variable number or distance or radius of the small sphere(s) employed as a representation of heterogeneous sub-regions within the large sphere.

This can be understood due to the use of modified Gaussian profiles for the SUV of all spheres which is characterized by their very smooth spatial changes. Nevertheless it can be observed from the results in Figure 4(Fig. 4) that the degree of heterogeneity gains the largest change when the number N of the small spheres at the periphery of large sphere increases from 1 to 4. It is also interesting to note that only the Contrast exhibits consistently large variations while the others show either no or very little changes among the GLCM parameters. Recall that the Contrast as defined in Eq. (5) is dominated by the probability of large gray level differences among the neighboring voxels in an input image. As a result the Contrast parameter provides a gauge similar to the H index and the two correlates quite strongly. While the increases of both H index and Contrast are largest for the case of rising N among the multi-sphere phantoms, however, the sources of the increase are different: the former is due to number of peripheral spheres and the latter is more related to the 2^nd^ power of the local gray level differences. Consequently the above results demonstrate that the H index defined by the new model can not only reduce the complexity of calculation involved in conventional image characterization methods such as GLCM but also provide an improved measure of global characteristics of tumor heterogeneity. The above conclusion is further corroborated by the results of analysis performed with the 2D phantoms as presented in Figure 2(Fig. 2) and Figure 5(Fig. 5). It is shown that the H index can characterize the image heterogeneity with higher sensitivity than the 4 GLCM parameters.

**Figure 6:**
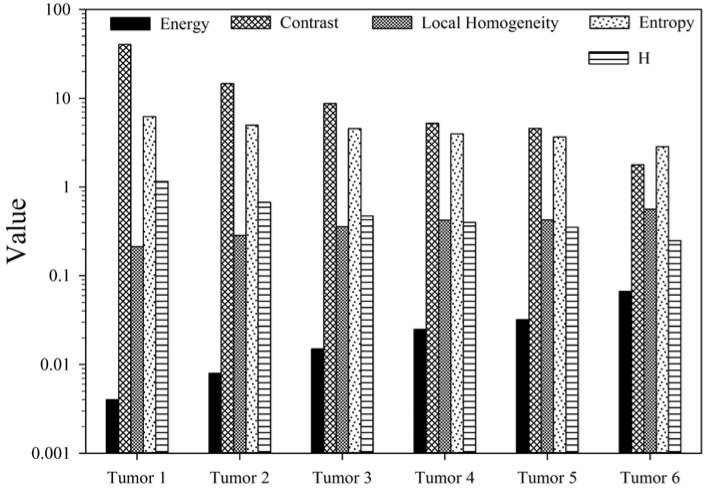
The GLCM parameters and H index obtained from six lung cancer patients' tumors, three of them are shown in Figure 3

A further comparison of the H index and GLCM parameters with the [18]F-FDG PET image data of the 6 patients in Figure 6(Fig. 6) confirms the conclusions presented above. Unlike the highly symmetric assemblies of the modified Gaussian spheres, the patients' images present high degree of heterogeneity and thus all of the GLCM parameters' values vary among the tumors. Still the H index, Contrast and Entropy parameters decrease from Tumor 1 to Tumor 6, indicating the tumor heterogeneity varies from Tumor 1 to 6 in a descending order. On the other hand, the Energy parameter increases from Tumor 1 to 6 and so does the Local Homogeneity. The opposite variations between Contrast and Local Homogeneity among the 6 tumors demonstrate that the co-occurrence probability of same or similar SUV among the neighboring voxels as characterized by the Local Homogeneity varies in an opposite fashion than that of significantly different SUV as characterized by the Contrast. These results show that the multiple GLCM parameters, only 4 are used here, can provide a comprehensive interpretation of the PET image data in terms of the statistical features of the image textures. The GLCM model, however, can be difficult to implement in clinics because of the abstract definitions and different sensitivities to tumor heterogeneity among the multiple parameters. In comparison, the H index yields an intuitive and fairly robust tool for extracting information on the spatial gradient of the tumor heterogeneity as demonstrated by our results. Moreover, the sign of H index provides additional information on relative changes in SUV between the SUV-based tumor center and periphery.

Many studies have shown that heterogeneity in intratumor metabolism plays an important role in tumor response to treatment (Kidd and Grigsby, 2008[10]; Tixier et al., 2011[16]). Our preliminary study with patient data has also shown this high correlation, which indicates the potentials of utilizing the H index in studying tumor response to radiation treatment. 

## Conclusion

We have developed a new model with a single-parameter H index for assessment of tumor heterogeneity from the image data of [18]F-FDG PET. With the phantoms and patients' image data we have shown that the H index allows characterization of differential SUV distribution in a tumor. A comparison of the H index and 4 GLCM parameters demonstrates that the new model has the capacity to extract the information on intratumor heterogeneity from the 2D and 3D image data and possesses the simplicity needed for potential clinical applications. The correlation of the H index with the pathological grading of the tumor, response to therapy and patient's prognosis, however, remains to be further investigated with large amounts of patient data. We are currently conducting a clinical study to answer these critical questions.

## Acknowledgements

The authors acknowledge the support of NSFC (grants #81171342, #81041107 and #31000784).

## Conflict of interests

The authors declare that there is no conflict of interests regarding the publication of this article.
